# Reinforcement of nylon 6,6/nylon 6,6 grafted nanodiamond composites by *in situ* reactive extrusion

**DOI:** 10.1038/srep37010

**Published:** 2016-11-14

**Authors:** Eun-Yeob Choi, Kiho Kim, Chang-Keun Kim, Eunah Kang

**Affiliations:** 1 School of Chemical Engineering and Material Science, Chung-Ang University, 221 Heukseok-Dong, Dongjak-Gu, Seoul, Korea

## Abstract

Nanodiamond (ND), an emerging new carbon material, was exploited to reinforce nylon 6,6 (PA66) polymer composites. Surface modified nanodiamonds with acyl chloride end groups were employed to chemically graft into PA66, enhancing the interfacial adhesion and thus the mechanical properties. The ND grafted PA66 (PA66-g-ND) reinforced PA66 composite prepared by *in situ* reactive extrusion exhibited increased tensile strength and modulus. The tensile strength and modulus of PA66/3 wt.% PA66-g-ND composites were enhanced by 11.6 and 20.8%, respectively when compared to those of the bare PA66 matrix. Even the PA66/pristine ND composites exhibited enhanced mechanical properties. The PA66-g-ND and the homogeneously dispersed PA66-g-ND in PA66 matrix were examined using X-ray photoelectron spectroscopy, thermogravimetric analysis, scanning electron microscopy and transmission electron microscopy techniques. The mechanical properties and thermal conductivities of the nanodiamond incorporated PA66 composites were also explored. The enhanced mechanical properties and thermal conductivities of the PA66-g-ND/PA66 composites make them potential materials for new applications as functional engineered thermoplastics.

Carbon based nanomaterials such as carbon nanotubes (CNT), fullerene, graphite, graphene nanosheets, and nanodiamonds (NDs) have attracted great attention due to their chemical and physical properties such as large surface areas, outstanding mechanical properties, easy access of surface functionality, etc.^1^,^2^. Especially, nanodiamonds have been widely used for electrical materials since NDs have promising properties such as energy absorbance and high capacitance. The applications of nanodiamonds include lubrication, electromagnetic shielding, special catalysts, polymer composite filler as other carbon materials, etc.^3^,^4^. Nanodiamonds can be produced by different methods such as, detonation, laser ablation, high-energy ball milling of diamond microcrystals under high pressure and high temperature, and plasma-assisted chemical vapor deposition [4]. However, the nanodiamonds obtained by these methods tend to form agglutinations of sizes 50–100 nm rather than single particles of 5 nm size, which hampers the nanoscale applications of the nanodiamonds^5^. To overcome this issue, deagglomeration of nanodiamond has been tried using methods like ceramic bead milling with additives, oxidation of ND, and laser chemistry^6^,^7^,^8^,^9^,^10^. These aggregates are known to be subjected to the Coulomb’s law by the Van der Waals interaction rather than the electrostatic interaction^9^,^11^. Moreover, facets with varied sizes create dipole moments which determine the ND agglutination size^12^. Agglutinated nanodiamonds are held by covalent carbon-carbon bonds between nanocrystals, forming unusually strong aggregates even under strong ultrasonication. Disintegrated nanodiamonds composed of agglutinats and small aggregates might be excellent composite materials with enhanced mechanical properties and interfacial adhesion.

Nanodiamonds have emerged as a new platform for nano/inorganic materials due to their interfacial integration on physical contact with the polymer composites. Several studies have reported the advantages of nanodiamonds as reinforcing enhancers in polymer composites^3^,^13^,^14^,^15^,^16^,^17^,^18^. It has been reported that high concentrations of NDs added to polyacrylonitrile (upto 80 wt.%) and polyamide 11 (40 wt.%) matrices fabricated by electrospinning resulted in 4 times increase in the Young’s modulus and 2 times increase in the hardness even at 20 wt.% nanodiamond in polyamide 11^15^. Crosslinkable NDs with amine end groups were embedded within an epoxy resin or maleic anhydride grafted high density polyethylene for enhancing the Young’s modulus and the hardness^3^,^16^. Nanodiamond dispersion seems to be critical for both simple physical ND mixing and chemical ND conjugation in polymer matrices in order to have an effect on the mechanical properties of the composites. In particular, surface-modifying capabilities found in charges and other functional groups on the ND substrate provide the potential for effective reinforcement. One advantage of employing the nanodiamonds as an interfacial enhancer in polymer composites comes from strong physical adsorption induced by the high surface-to-volume ratio and the large contact surface area with different sized facets. Large contact surface area of nanodiamonds may provide enhanced adhesion between the polymer matrix and the NDs thereby ensuring the reinforcement of the polymer composites. The chemically inert ND surface in the polymer composites improves the mechanical properties. Moreover, the unusually stable nanodiamond agglutination is suitable to overcome the extrusion process while the length of the carbon nanotube with high aspect ratio is reduced, which may act as defects in long term estimation^19^. Even though several studies promise nanodiamonds to be a new source of composite materials, classified and elemental cases require extensive investigation for the development of engineered thermoplastics.

In specific, Polyamide 66 (PA66) with the advantages of high toughness, abrasion resistance, low density and a low frictional coefficient has found versatile applications such as tire cords, ropes and airbags. Moreover, PA66 is often employed to automobile parts to reduce the car weight because the use of hybrid energy aims at the high energy efficiency. Thus, PA66 composites with glass fiber^20^,^21^, talc^22^, clay^23^ or carbon materials have been employed to fortify the stiffness and the strength. Reinforced PA66 with new carbon materials can further offer new applications such as functionally engineered thermoplastics. The success key of PA66 composites is the critical improvement in the interfacial adhesion between the nanoscale reinforcing fillers and the main polymer matrix. Enhanced interfacial adhesion of PA66 has been achieved with covalent bonding^19^ or physical adsorption of pyrene via π-π stacking on the CNT surface^24^. CNT functionalization with physical adsorption of naphthalene containing copolymer was also introduced into the PA66 matrix to enhance the CNT dispersion^25^. Overall, the PA66 composite with functionalized inorganic CNTs showed improved mechanical properties compared to those with inert CNTs or nylon 6,6 only.

This study examines the potential reinforcement of PA66 by the addition of nanodiamonds. The dispersion of the chemically modified functional nanodiamonds and their effect on the mechanical properties of the PA66/ND composites were investigated. The new carbon materials, nanodiamonds and surface functionalized nanodiamonds with acyl chloride were introduced to the PA66 matrices. The PA66 composite with the nanodiamonds was produced by reactive *in situ* extrusion using a twin extruder. Functionalized nanodiamonds with acyl chloride were allowed to chemically conjugate with the amine end group of PA66 during melt mixing, and as a result, PA66 grafted NDs (PA66-g-ND) were fabricated (Fig. 1a). The chemical functionalization of the nanodiamonds and the PA66-g-ND was characterized using Fourier transform infrared spectroscopy (FTIR), X-ray photoelectron spectroscopy (XPS), thermogravimetric analysis (TGA) and transmission electron microscopy (TEM). The mechanical properties and the thermal conductivities of the composites of PA66/ PA66-g-ND were also studied.

## Results and Discussion

### Characterization of ND-COCl and PA66-g-ND

**Figure 1:**
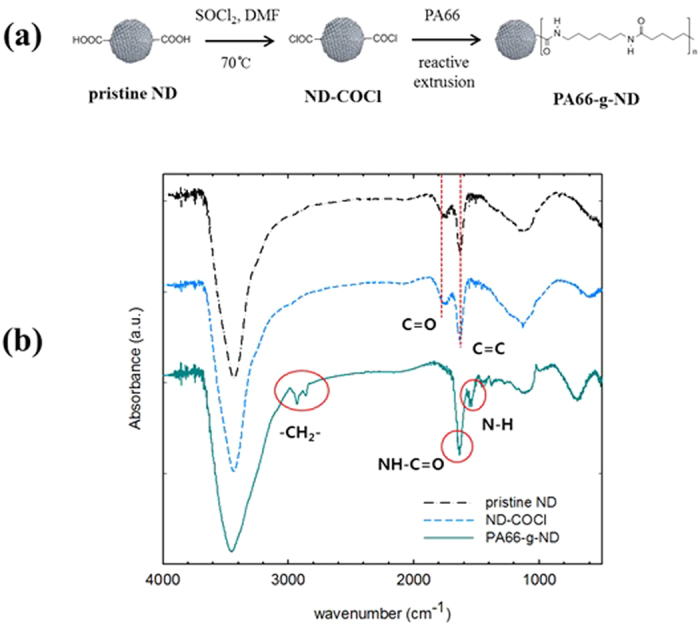
(**a**) Schematic diagram showing the synthesis of PA66-g-ND by reactive extrusion, and (**b**) FT-IR spectra of the pristine NDs, ND-COCl and PA66-g-ND.

Figure 1b shows the FTIR spectra of the pristine ND, ND-COCl and PA66-g-NDs. As shown in Fig. 1b, the spectra of the pristine ND and ND-COCl exhibited stretching peaks at 1750 and 1630 cm^−1^, which originate from the carbonyl groups and the C=C sp^2^ hybridized bonds, respectively. The FTIR spectrum of the PA66-g-ND showed the stretching peaks of the methylene groups (2850–3000 cm^−1^) and N-H groups in amide (1550 cm^−1^), corresponding to those of PA66. In particular, the stretching peak at 1630 cm^−1^ in the FT-IR spectrum of PA66-g-ND exhibited strong intensity, compared to that of the pristine NDs and NDs-COCl. It is believed that the stretching of the amide groups occurred at approximately 1630–1680 cm^−1 ^26 and may overlap with the stretching of the C=C sp^2^ hybridized bonds.

**Figure 2:**
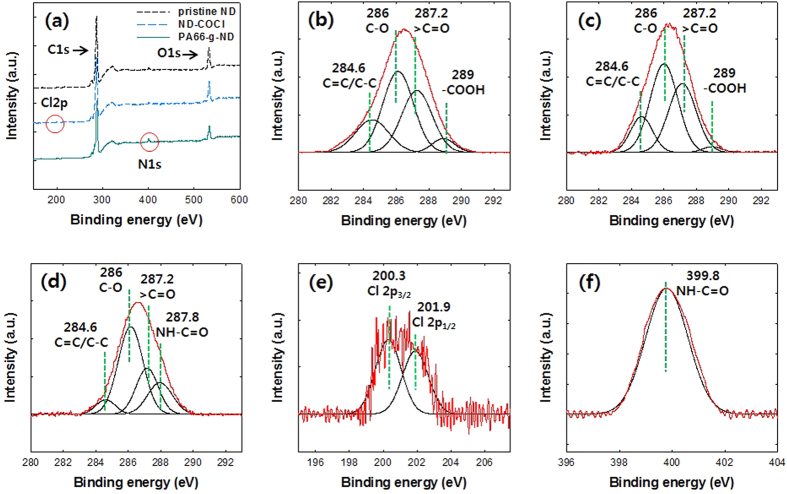
XPS scan spectra of NDs: (**a**) wide scan spectra of pristine NDs, ND-COCl and PA66-g-ND, deconvoluted spectra of (**b**) C 1 s for the pristine NDs, (**c**) C1s for the ND-COCl, (**d**) C1s for the PA66-g-ND, (**e**) Cl 2p for the ND-COCl and (**f** ) N 1 s for the PA66-g-ND.

XPS analyses were also performed to confirm the functional surface in ND-COCl and the graft formation in PA66-g-ND. Figure 2a shows the XPS wide scan spectra of the pristine ND, ND-COCl and PA66-g-ND. The C 1 s and O 1 s peaks were observed in the wide scan spectra of both the pristine ND and ND-COCl, while the Cl 2p peak was observed only in the wide scan spectra of ND-COCl. The N 1 s peak appeared in the wide scan spectra of PA66-g-ND because the amide groups were formed in the PA66 grafted on the surface of nanodiamonds from the conjugation between the amine end group of PA66 and the carboxyl group of the nanodiamonds. The highly resolved peaks of Cl 2p and N 1 s peaks between pristine ND, ND-COCl and PA66-g-ND was provided in Figure S1 in the Supplementary Information.

Deconvolution of the respective peaks into the chemical component peaks was carried using Gaussian fitting. The deconvoluted peaks of the pristine NDs, ND-COCl and PA66-g-ND are shown in Fig. 2b–e. As shown in Fig. 2b,c, the C 1 s peaks of the pristine ND and ND-COCl were deconvoluted into four binding energies: 284.6 (-C=C-), 286.0 (-C-O-), 287.2 (>C=O) and 289 eV (-COOH). The deconvoluted peak at 289.0 eV in ND-COCl exhibited a weak intensity, compared to that of the pristine NDs. This indicates that the acyl chloride group of the ND-COCl was formed from the carboxyl groups on the pristine NDs after reaction. The C 1 s peak of the PA66-g-ND (Fig. 2d) was also deconvoluted into four binding energies at 284.6 (-C=C-), 286 (-C-O-/-C-N-), 287.2 (>C=O) and 287.8 eV (NH-C=O). The C 1 s peak of PA66-g-ND showed a shoulder at the center of the peak at 287.8 eV due to the amide bond formation. The deconvoluted peak at 287.8 eV in PA66-g-ND showed a strong intensity. The deconvoluted peaks corresponding to -C-N- and NH-C=O indicated that PA66 was successfully grafted onto the NDs. The Cl 2p peak of ND-COCl (Fig. 2e) was curve-fit with two component peaks of Cl 2p_1/2_ and Cl 2p_3/2_. The peaks observed at binding energies of 200.3 and 201.9 eV were assigned to Cl 2p_1/2_ and Cl 2p_3/2_, respectively. The presence of Cl 2p_1/2_ and Cl 2p_3/2_ peaks are attributed to the C-Cl bonds of ND-COCl. The N 1 s peaks of the PA66-g-NDs (Fig. 2f) were fitted with a single component peak. The binding energy of peak at 399.8 eV was originated from the amide bond in PA66-g-ND, indicating that the amide bond was formed between the PA66 and ND-COCl. In summary, the intensities and the binding energies observed in the C 1 s and N 1 s spectra confirmed the formation of the PA66-g-ND during the reactive extrusion.

**Figure 3:**
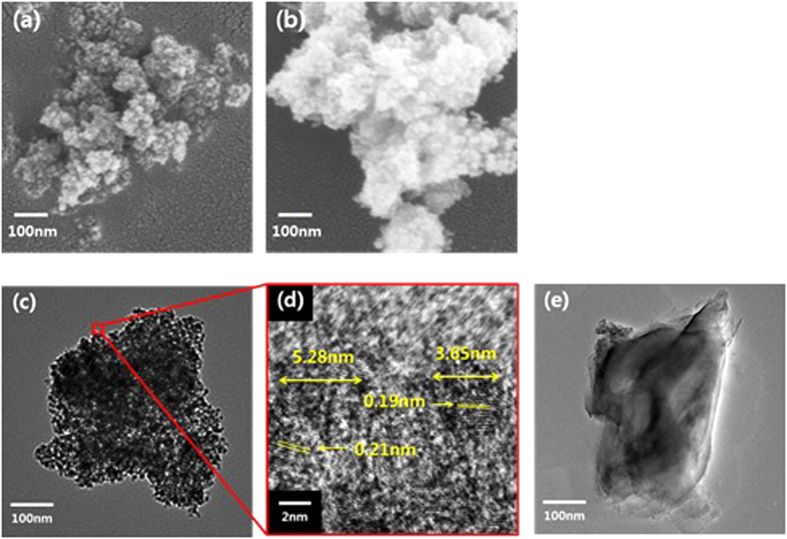
The FESEM images of the (**a**) pristine NDs and (**b**) PA66-g-ND, and HR-TEM micrographs of the (**c**) pristine NDs, (**d**) pristine NDs in high resolution and (**e**) PA66-g-ND.

Figure 3 shows the FESEM and the TEM images of the pristine NDs and the PA66-g-ND. The PA66-g-ND was prepared by the removal of the unreacted PA66 after reactive extrusion. As shown in Fig. 3a,b, a significant change of contrast of PA66-g-ND was observed, compared to the pristine NDs. The surface of the PA66-g-NDs became rough and individual nanodiamonds were not seen because the nanodiamond surface was covered with the grafted PA66. Detailed examination of the surface was carried out using TEM and the images are shown in Fig. 3c,d. The drops of the nanodiamond dispersion in formic acid were placed on the TEM grid and dried at 80 °C. Pristine NDs existed in the form of aggregates of several hundred nanometers without disassociation into individual nanodiamond crystallites. The magnified image of the pristine nanodiamonds showed that each crystalline structure was 4–5 nm in size (Fig. 3d) and the carbon crystalline arrangement was observed with a spacing of 0.2 nm. Nanodiamonds existed as adherent form with facet to facet. The TEM image of the PA66-g-NDs showed a thin film on the nanodiamond aggregates, indicating graft formation of PA66 onto ND surface (Fig. 3e). Since the nanodiamond aggregates were stable, a sheet-like thin film formed from the PA66 graft was enveloped on the ND aggregates. It indicates that a conjugation has occurred between the acyl chloride on the nanodiamonds and the amine end group in the PA66.

**Figure 4:**
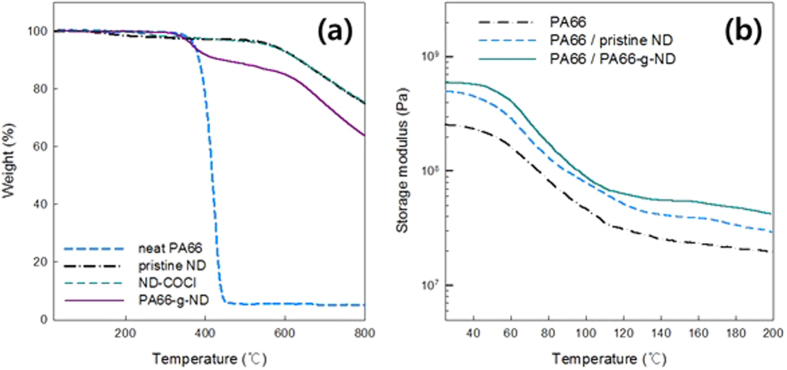
(**a**) The TGA thermograms of the PA66, pristine ND, ND-COCl and PA66-g-ND, (**b**) The storage moduli of the PA66 and PA66 composites containing 1wt.% of NDs observed using DMA.

TGA of the pristine NDs, ND-COCl, and PA66-g-NDs allows measurement of the quantitative grafting amount. TGA thermograms indicating the inertness of the pristine NDs, and quantitative PA66 grafting amounts are presented in Fig. 4a. The pristine NDs and NDs-COCl underwent thermal degradation at 540 °C, similar to the previous studies^27^. The thermal degradation of the nanodiamonds at 540 °C implied that a graphene-like amorphous layer existed on the nanodiamond surface. The oxidation of the nanodiamonds occurred at 540 °C with the removal of the amorphous graphene-like layer^28^. In contrast, the thermogram of the PA66 presented thermal degradation starting at about 360 °C. From the thermograms of the pristine ND, ND-COCl, and PA66, the thermal degradation of PA66-g-ND was found to be approximately 10.3 wt.%. The mass loss of 10.3 wt.% indicated the amount of PA66 grafted onto the nanodiamonds.

### Characteristics of the PA66/ND composites

The storage moduli of the PA66 and PA66 composites containing 1 wt.% of the NDs were obtained from DMA as a function of temperature and is shown in Fig. 4b. The storage modulus of the ND introduced PA66 composites are constantly higher in the range of the experimental temperature, compared to that of the bare PA66. The introduction of the pristine NDs and PA66-g-NDs increased the storage modulus values, compared to that of the bare PA66. Loss modulus at 1 Hz for PA66, PA66/pristine NDs and PA66/1 wt.% PA66-g-NDs appears at 63.5, 69.5, and 68.5 °C, respectively. Loss moduli of PA66, PA66/pristine NDs and PA66/1 wt.% PA66-g-NDs are 1.91 × 10^7^, 3.96 × 10^7^, and 4.20 × 10^7^ Pa, respectively. Loss modulus (E″) indicates the viscous response of the composite and the energy dissipation when a material deforms. Increased loss modulus and storage modulus with the incorporation of the pristine ND suggests that mechanical stress was dissipated from the polymer matrix in to the nanodiamond agglutinations. The surface modified ND-COCl augmented the storage and loss modulus in the PA66/1 wt.% PA66-g-NDs composites than those in the PA66/pristine ND. The Chemically grafted nanodiamonds reinforced the mechanical properties due to the uniformly dispersed grafted ND in the PA66 matrix.

**Figure 5:**
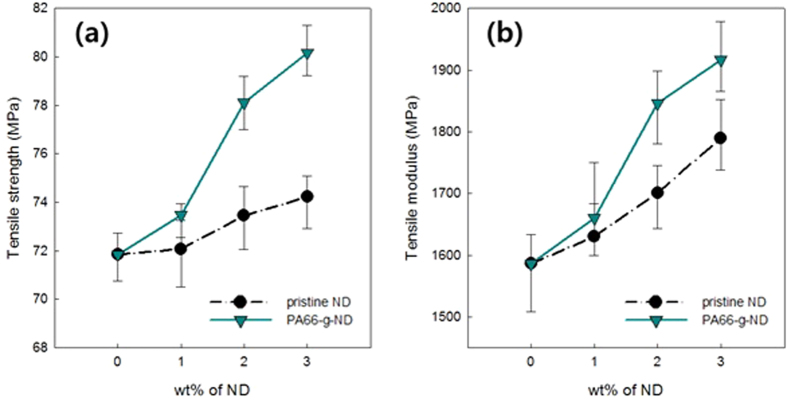
The changes in the mechanical properties of the PA66 composites with the addition of the pristine NDs or PA66-g-ND as represented by the (**a**) tensile strength and (**b**) tensile modulus, as a function of nanodiamond composition in the PA66 composites.

The influence of nanodiamonds on the mechanical properties of the PA66 composites was studied with increased contents of pristine ND or PA66-g-ND. Figure 5 shows the reinforcement in the tensile strength (Fig. 5a) and the tensile modulus (Fig. 5b) of the PA66 composites that were obtained by reactive extrusion. The composition of the pristine ND or the surface modified ND-COCl was increased from 0 to 3 wt.%. Both the tensile strength and the modulus of the composites were enhanced with increase in the contents of pristine ND or PA66-g-ND up to 3 wt.%. The tensile strength of the PA66 composite with 3 wt.% addition of pristine NDs increased to 74.2 MPa, compared to that of bare PA66 (71.9 MPa). The tensile modulus of PA 66 composite with 3 wt.% addition of pristine NDs also showed improvement to 1.79 GPa, compared to that of bare PA66 (1.59 GPa). These results showed reinforcement of 3.3% in the tensile strength and 12.8% in the tensile modulus with addition of even the pristine NDs. It is notable that the addition of 1 wt.% of the pristine ND increased the tensile modulus by 4.7%. The tensile strength of the PA66 composite with 3 wt.% addition of the PA66-g-ND was increased to 80.2 MPa. The tensile modulus of the PA66 composite with 3 wt.% addition of the PA66-g-NDs was enhanced to 1.92 GPa. Overall, a reinforcement of 11.6% in the tensile strength and 20.8% in the tensile modulus was obtained from the addition of 3 wt.% of the PA66-g-ND. For composites incorporating the same composition of NDs, the tensile strength and the modulus of the PA66/PA66-g-ND composite were significantly improved within probability. p < 0.01. The tensile moduli and strengths at 3 wt.% of PA66-g-ND composition were critically reinforced by 9.1% and 15.4%, respectively compared to the values at 1 wt.% PA66-g-ND. With the increased ND content, the composites were reinforced, showing both enhanced tensile strength and modulus for pristine NDs or PA66-g-ND. At a constant ND content, the tensile moduli and strengths of the PA66 composite followed the order: PA66 < pristine ND < PA66-g-ND. Mechanical enhancement conducted by *in situ* reactive nanodiamond onto PA66 was compared with previous studies, as shown in Table 1. Most inorganic fillers were generally added with small composition from 0.1% to 5% except graphene. From the comparison, *in situ* reactive nanodiamonds is good candidate as a filler for mechanical enhancement. Another, the plasticity of PA66/PA66-g-ND composite composites was significantly enhanced, compared to PA66/ND composites in the stress-strain curves (Figure S2). The increased plastic region for PA66/PA66-g-ND composite indicates that improved interfacial adhesion and uniform dispersion of PA66-g-NDs in PA66 matrix prevent dramatic decrease in strain, maximizing their plasticity.

The melt mixing processing using extruder is conventional method to prepare pelletized composite materials. Physical contacts between fillers and polymeric matrix are limited to control since shear stress and temperature are relatively established in melt mixing processing. Formerly prepared chemical modification of ND surface with highly reactive acyl chloride provides the chance to be integrated into PA66 matrix, forming PA66-g-ND. ND filler with acyl chloride provided effective methods, inducing enhanced interfacial adhesion during the melt mixing process.

**Figure 6:**
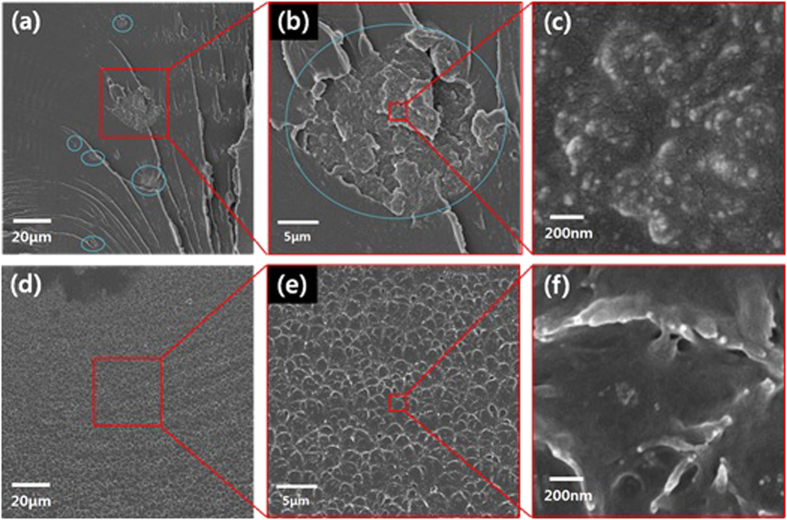
Cross-sectional morphologies of the fractured PA66 composites containing 1 wt.% of (**a**–**c**) pristine NDs and (**d**–**f**) PA66-g-ND, obtained using FESEM.

The cross sections of the fractured PA66 composites were investigated using FESEM. The ND dispersion and the state of interfacial adhesion between the ND aggregates and the PA66 matrices were examined by studying the morphologies of the fractured cross-section of PA66 composites. Figure 6 shows that the pristine NDs in the PA66/pristine ND composites are not homogeneously dispersed and aggregated on the microscale. The pristine NDs in nanodiamond rich region existed inwith the form of large aggregates (blue circles in Fig. 6a,b). This indicates that the residual time of approximately 5 min in the twin extruder might not be enough to physically disperse the pristine ND at the nanoscale. In general, the nanodiamonds are known to be present in the form of agglutinates of size 50 nm or aggregates of several hundred nanometers in diameter. Even under strong ultrasonication, the NDs are difficult to be separated into individual ND nanocrystals (5 nm) due to the forces of strong Van der Waals bond, electrostatic interaction and dipole moment generated from facet to facet^9^,^11^. The strong interaction within the nanodiamonds may act as a strong focal region to reinforce polymer composites, supporting the improved tensile modulus and strength in the PA66/pristine NDs^29^,^30^.

In contrast, the PA66-g-NDs are uniformly dispersed in the PA66 composites as shown in Fig. 6d,e. From the high-magnification images of PA66/PA66-g-ND composites (Fig. 6d,e), it is clear that homogeneously dispersed PA66-g-NDs were present at the fracture surface and the interfaces between the PA66 matrix, and PA66-g-ND had good adhesion without any fissures. The results indicate that the preparation of PA66-g-NDs by reactive extrusion was effective in reinforcing the PA66, as surface modified NDs-COCl was introduced into the PA66 matrix through the extrusion process, which assisted the homogeneous dispersion in the PA66 matrix. These findings well correspond to the results of mechanical properties of the PA66/PA66-g-ND composites.

Table 2 presents the thermal diffusivity, specific heat capacity and calibrated thermal conductivity of the PA66 composites at various contents of nanodiamonds. Thermal diffusivity of PA66 and ND composites did not show significant difference between PA66 and ND-incorporated PA66 composites regardless of the increase of ND concentration. Measured specific heat capacities of PA66 and pristine ND were 1.667 and 10.854 (J/g·K), respectively. Calibrated specific heat capacities with weight ratio of PA66 and nanodiamond were used for thermal conductivity. The specific heat capacity of the ND/PA66 composites showed a gradual increase, compared to the bare PA66. The higher specific heat capacity indicates that higher amount of heat can be absorbed by the ND/PA66 nanocomposites than by bare PA66 at the same temperature difference. In contrast, the thermal diffusivity of the PA66/ND composites decreases upon addition of NDs. The thermal resistance between the PA66/ND matrix and large contact resistance between NDs, like other carbon-based materials^34^,^35^, can limit heat flow in the PA66/ND composites. The enhanced thermal conductivity of the ND-containing PA66 was contributed by the specific heat capacity, rather than the thermal diffusivity. It indicates that the dispersion, adhesion, and interaction of the nanodiamond in the PA66 matrix might determine the final thermal conductivity^13^,^36^,^37^.

## Conclusions

Emerging new carbon materials, nanodiamonds were explored to reinforce PA66 polymer composites. Surface modified ND-COCl was employed to enhance the interfacial adhesion and to strengthen the mechanical properties. The formation of PA66-g-ND during the reactive extrusion reinforced the PA66 composite, proved by the enhanced tensile strength and modulus. The tensile strength and tensile modulus of the PA66/PA66-g-ND (3 wt.%) composites were augmented by 3.2 and 12.6%, respectively, compared to those of the bare PA66 matrix. Even the pristine NDs/PA66 composite displayed enhanced mechanical properties. The formation of PA66-g-ND and the homogeneously dispersed PA66-g-ND in the PA66 matrix were examined using XPS, TGA, SEM and TEM. Moreover, the introduction of nanodiamonds into the PA66 matrix enhanced the thermal conductivity. The improved mechanical properties and the enhanced thermal conductivity of PA66-g-ND/PA66 composites make them potential materials as functional engineered thermoplastics interfacing organic and electrical materials.

## Materials and Procedure

### Materials

Commercial grade PA66 was obtained from LG Chemicals (Seoul, Republic of Korea). According to the characterization of the commercial grade PA66, the molecular weight, M_w_ was 43,000 g/mol and the number average molecular weight, M_n_ was 22,000 g/mol, as determined by gel permeation chromatography (GPC) using polystyrene standards. The nanodiamonds of G2 grade were procured from Plasma Chem (Berlin, Germany). Formic acid (HCOOH, reagent grade), used as a PA66 dissolving solvent, was purchased from Aldrich Chemicals (Milwaukee, WI, USA). Thionyl chloride (SOCl_2_) and dimethylformamide (DMF) were also purchased from Aldrich Chemicals (Milwaukee, WI, USA). PA66 was dehydrated under vacuum at 100 °C for 24 h before use.

### Preparation of ND-COCl and PA66-g-ND

The synthesis procedure for PA66-g-ND is shown in Fig. 1a. The nanodiamonds were used as received for the bond formation of acyl chloride on the surface. The XPS results were consistent with the G2 grade nanodiamonds and the acid washed nanodiamonds (see Figure S3 and Table S1 in the Supplementary Information). For further chemical modification of ND surface, ND (G2 grade) was used as received. In order to obtain the functionalized NDs with acyl chloride groups, the pristine NDs (5 g) were dispersed in SOCl_2_ (200 ml) under sonication for 30 min until nanodiamond aggregation was not visible to the eye. The nanodiamond dispersion was continuously stirred under nitrogen purging for 1 h and DMF (1 ml) was added as a catalyst for the acyl chloride formation. The reaction of ND dispersion in SOCl_2_ was maintained at 70 °C for 24 h. The resulting ND dispersion with acyl chloride was vacuum-filtered through a nylon membrane (450-nm, Watman, Maidstone, UK). The filtered NDs were washed with tetrahydrofuran (3 × 200 ml) and dried for 24 h in a vacuum oven at room temperature (hereafter referred as “ND-COCl”).

The PA66/PA66-g-ND composites were prepared from PA66 and ND-COCl by reactive extrusion. The reactive extrusion was carried out with the change of of ND-COCl concentration, using a twin extruder (BA-11, L/D ratio ¼ 40, Bau Technology, Seoul, Korea). The respective temperatures of the twin extruder were 260 °C at the feeding zone, 280 °C at the melting zone, 290 °C at the mixing zone and 280 °C at the exit die. The material feed rate and the extrusion speed were maintained constant at 10 g/min and 300 rpm, respectively. The conjugation between the acyl chloride on the nanodiamond surface and the amine end groups of PA66 resulted in the formation of PA66-g-ND during the extrusion. The extruded PA66/PA66-g-ND composite was immediately quenched in a water bath after extrusion, pelleted and dried at 80 °C for 24 h. Subsequently, 1 g of the PA66/ PA66-g-ND composite was dissolved in formic acid (300 ml) to remove the unreacted PA66. The dispersion of PA66-g-ND and dissolved PA66 was separated using a centrifuge at 10,000 rpm for 30 min. The supernatant solution was removed and the PA66-g-ND at the bottom was collected. The collected PA66-g-NDs were again dispersed in formic acid (300 ml). The same procedure of washing was repeated five times for complete removal of the ungrafted PA66. The resulting product was dried for 24 h in a vacuum oven at 80 °C.

### Characterization of ND-COCl and PA66-g-ND

The chemical characteristics of pristine ND, ND-COCl, and PA66-g-ND were examined by FTIR (Magna 750, Nicolet, WI, USA) analysis and XPS (VG Microtech, ESCA2000, UK). IR spectra were collected over 30 scans in the 4000–500 cm^−1^ region using attenuated total reflection mode with a resolution of 4 cm^−1^. XPS(was equipped with a spectrometer with a Mg Kα X-ray source (1253.6 eV) and a hemispherical analyzer. The XPS spectra were obtained in high-resolution mode with 20 eV pass energy and a 0.1 eV step size. All the binding energies were calibrated using the standard carbon (C 1 s) peak at 284.5 eV. For curve fitting, the widths of the Gaussian peaks were kept constant in each spectrum. Field emission scanning electron microscopy (FESEM, model: Sigma, Carl Zeiss, Germany) and high resolution transmission electron microscopy (HRTEM, model: JEM 2000EXII, JEOL, Japan) were employed to investigate the morphologies of the pristine NDs, NDs-COCl, PA66-g-NDs and composites. The samples for FE-SEM were prepared with 0.5–3 nm thick platinum coating using a high resolution sputter coater (model: SPT-20, COXEM, Korea). HRTEM grids were prepared by placing dispersions of ND in formic acid onto rectangular mesh grids and drying in a vacuum oven at 80 °C until most of the formic acid had evaporated. TGA (model:TGA-2050, TA Instruments, USA) of NDs, NDs-COCl, and PA66-g-NDs was performed under nitrogen at a heating rate of 10 °C/min. The specimens for the TGA experiments were dried in a vacuum oven at 80 °C for a day.

### Characterization of the PA66/PA66-g-ND composites

The PA66/ND composites were prepared with various ratios of ND-COCl (0, 1, 2 and 3 wt.%) by reactive extrusion and were dried in a vacuum oven at 80 °C for a day before use. The specimens for the tensile strength measurements and dynamic mechanical analyses were prepared by compression molding. The pellets were poured into a mold in a compression molding machine (model 25–12; Carver, Inc., USA) operating at a plate temperature of 280 °C and a holding pressure of 12 MPa. The melted composite was kept at 280 °C for 5 min, extruded into the shaped mold, and then cooled to room temperature for 2 h by natural convection. After being molded, the specimens were placed in a vacuum oven at 30 °C prior to the mechanical testing. The specimens for tensile testing were prepared in accordance with American Standards Testing Method (ASTM) specification D 638. The tensile tests were carried out using a universal testing machine (UTM-301, R&B Corp, Daejon, Korea) at a cross-head speed of 5 mm/min. The reported tensile property values represent the average values of five measurements. The storage moduli (E) of the PA66 and its composites were measured by dynamic mechanical analysis (DMA, SS6100, Seico Instruments, Japan) at a constant frequency of 1 Hz. The specimens for DMA were heated from 25 to 200 °C at a rate of 5 °C/min under nitrogen purging.

Composite films were also prepared by solvent casting from formic acid to examine the effects of the functionalized ND-COCl surface on the nanodiamond dispersion in the PA66 matrix. PA66 (5 g) was dissolved in formic acid (100 ml), and 1 wt.% of NDs and NDs-COCl were dispersed in the PA66 solution under sonication. Solutions cast onto a glass plate were dried in an oven at 30 °C for 6 h until most of the solvent had evaporated. The resulting films were finally dried for 24 h in a vacuum oven at 80 °C. Later, the morphologies were characterized using FESEM.

The thermal conductivities of the composites were calculated at room temperature using the following equation:


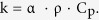


where, k, α, ρ and C_p_ represent the thermal conductivity (W/(m **·** K)), thermal diffusivity (mm^2^/s), density (g/cm^3^) and specific heat capacity (J/(g **·** K)) of the composite, respectively. The thermal diffusivities of all the films were measured by laser flash analysis using a Netzsch 447 nanoflash (NETZSCH, Selb, Germany). The specific heat capacity (C_p_) of the samples was examined by using differential scanning calorimetry (DSC, Netzsch DSC 200F3). The samples were heated from −20 to 100 °C at a heating rate of 3.0 °C/min, and the values of specific heat capacity at room temperature were obtained for the determination of thermal conductivity. The densities of the samples were determined by the water displacement method.

## Additional Information

**How to cite this article**: Choi, E.-Y. *et al.* Reinforcement of nylon 6,6/nylon 6,6 grafted nanodiamond composites by *in situ* reactive extrusion. *Sci. Rep.* **6**, 37010; doi: 10.1038/srep37010 (2016).

**Publisher’s note:** Springer Nature remains neutral with regard to jurisdictional claims in published maps and institutional affiliations.

## Supplementary Material

Supplementary Information

## Figures and Tables

**Table 1:** The comparison of mechanical reinforcement of polymer composites with various types of filler.

Authors	Type of filler	polymer	Content (wt. %)	Degree of reinforcement
Choi, E. Y. *et al.*^24^	Multi-walled carbon nanotube	PA66	1.0	5.8% (for tensile modulus)
Kim, K. T. *et al.*^25^	Multi-walled carbon nanotube	PA6	0.1	35.7% (for tensile modulus)
			0.5	12.1% (for tensile modulus)
			1.0	3.3% (for tensile modulus)
Wahit, M. U. *et al.*^31^	Organoclay	PA6/PP	4.0	~24% (for tensile strength)
Peng, B. *et al.*^32^	SiO_2_	PP	1.0	~15% (for strength at break)
			2.0	~20% (for strength at break)
			4.0	~18% (for strength at break)
Lu, C. T. *et al.*^33^	Graphene	HDPE	4.0	~8% (for elastic modulus)
			8.0	~15% (for elastic modulus)
			12.0	~24% (for elastic modulus)
Our study	pristine ND	PA66	3.0	12.8% (for tensile modulus)
	PA66-g-ND		3.0	20.8% (for tensile modulus)

**Table 2:** Thermal conductivities of the PA66 composites as nanodiamond contents.

	NDs (wt %)	Thermal diffusivity (mm^2^/s)	Specific heat capacity [J/(g · K)]	Thermal conductivity [W/(m · K)]
pristine ND	0	0.163 ± 0.010	1.667 ± 0.058	0.337 ± 0.032
	1	0.166 ± 0.011	1.759 ± 0.108	0.362 ± 0.046
	2	0.161 ± 0.009	1.851 ± 0.095	0.369 ± 0.039
	3	0.155 ± 0.001	1.943 ± 0.067	0.373 ± 0.015
PA66-g-ND	1	0.161 ± 0.002	1.773 ± 0.079	0.354 ± 0.020
	2	0.160 ± 0.004	1.879 ± 0.161	0.370 ± 0.040
	3	0.157 ± 0.001	1.985 ± 0.123	0.386 ± 0.026
