# Supplementary material for: Reinforcement of nylon 6,6/nylon 6,6 grafted nanodiamond composites by in situ reactive extrusion
Source: Sci Rep. 2016 Nov 14;6:37010. doi: 10.1038/srep37010 (PMC5107966; doi:10.1038/srep37010)
Supplement: Supplementary Information [file srep37010-s1.doc]

**Reinforcement of nylon 6,6/nylon 6,6 grafted nanodiamond composites via *in situ* reactive extrusion**

Eun-Yeop Choi^1^, Kiho Kim^1^ Chang-Keun Kim^1^,*, Eunah Kang^1^,*

^1^School of Chemical Engineering and Material Science, Chung-Ang University, 221 Heukseok-Dong, Dongjak-Gu, Seoul, Korea

*Corresponding author. Tel: +822 8205324, E-mail address: ckkim@cau.ac.kr

Tel: +822 8206684, E-mail address: eakangek@cau.ac.kr

*Highly resolved XPS Peaks of Cl 2p and N 1s of NDs*


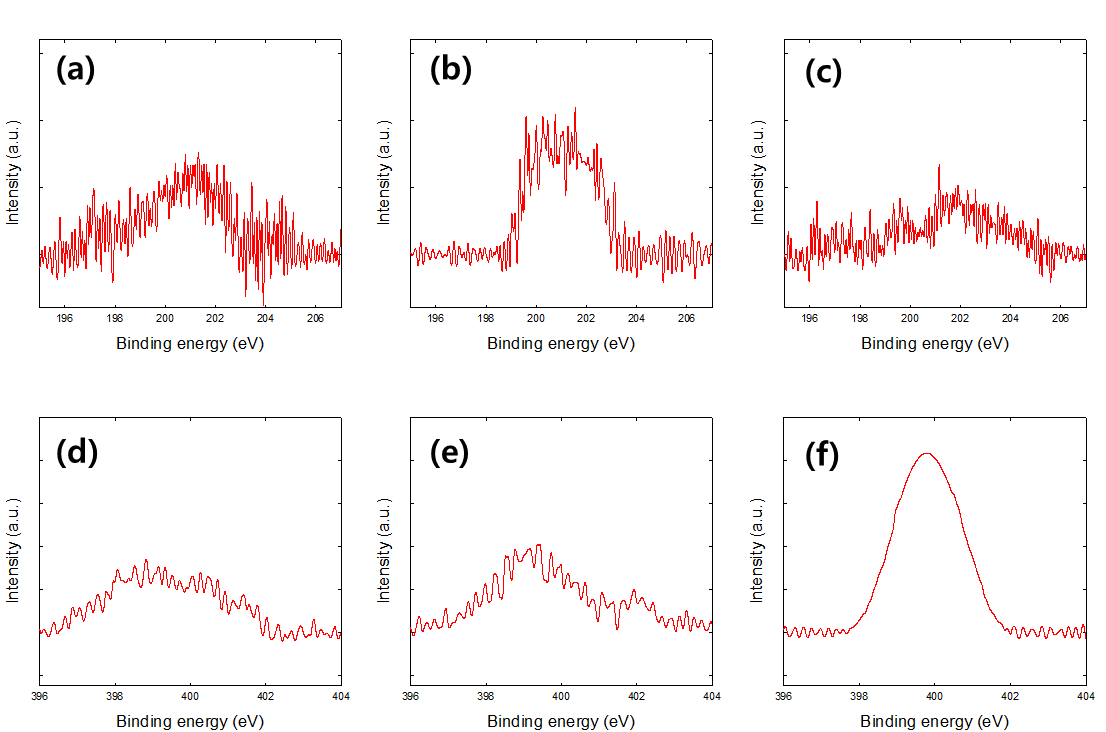


Figure S1. High-resolution Cl 2p XPS spectra of (a) pristine NDs, (b) NDs-COCl and (c) PA66-g-NDs, and high-resolution N 1s XPS spectra of (d) pristine NDs, (e) NDs-COCl and (f) PA66-g-NDs.

*Plasticity of PA66/ND composites*

Figure S3 shows stress-strain curves of the PA66/ND composites. The tensile modulus was calculated according to the following equation:

E = σ × L_0_ / (L – L_0_) = σ × 100 / ε_y_

where σ is tensile strength (yield strength), L_0_ is the length before stretching, L is the length after stretching, and ε_y_ is strain at yield point. The strain of the composites decreases with increasing ND content in both composites added with pristine ND and ND-COCl. For PA66/pristine ND composites, the dramatic decrease in the strain was observed with shorted plastic region as ND content reached at 3wt%. However, the strain of the PA66/PA66-g-ND composites was enhanced, compared to those of PA66/pristine ND composites at the same ND content. The loner plastic region the PA66/PA66-g-ND composites were also maintained. This result indicates that improved interfacial adhesion and uniform dispersion of PA66-g-NDs in PA66 matrix prevent dramatic decrease in strain and preserve their plasticity.


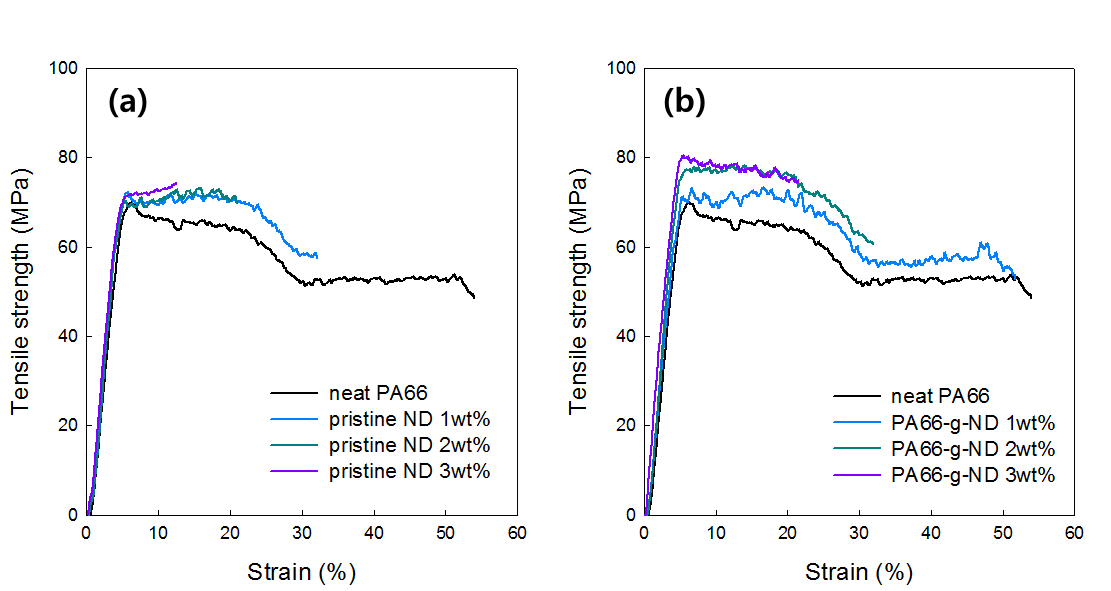


Figure S2. Stress-strain curves of (a) PA66/pristine ND composites and (b) PA66/PA66-g-*ND composites.*

*The XPS spectra of Pristine ND and acid-washed ND*


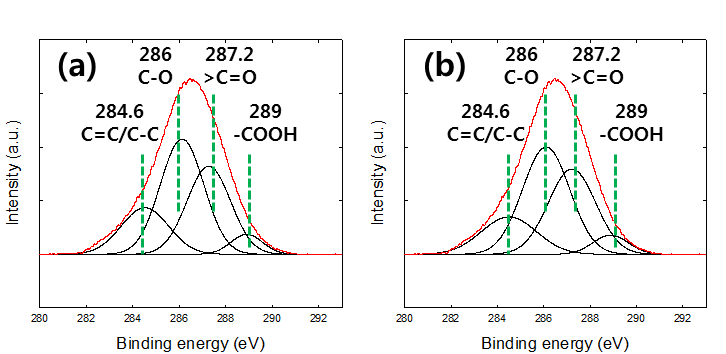


Figure S3. XPS scan spectra of (a) C1s curve fitting for the pristine NDs and (b) C1s curve fitting for NDs-COOH

Table S1. Chemical composition of the pristine NDs and NDs-COOH.

|  | C1s | O1s | Cl2p | N1s |
| --- | --- | --- | --- | --- |
| pristine NDs | 90.8 | 9.2 | - | - |
| NDs-COOH | 91.0 | 9.0 | - | - |

**References**

1 Choi, E. Y., Roh, S. C. & Kim, C. K. Noncovalent functionalization of multi-walled carbon nanotubes with pyrene-linked nylon66 for high performance nylon66/multi-walled carbon nanotube composites. *Carbon* **72** (2014).

2 Kim, K. T. & Jo, W. H. Non-destructive functionalization of multi-walled carbon nanotubes with naphthalene-containing polymer for high performance Nylon66/multi-walled carbon nanotube composites. *Carbon* **49**, 819-826 (2011).

3 Wahit, M. U., Hassan, A., Ishak, Z. A. M., Rahmat, A. R. & Othman, N. The effect of rubber type and rubber functionality on the morphological and mechanical properties of rubber-toughened polyamide 6/polypropylene nanocomposites. *Polym J* **38**, 767-780 (2006).

4 Peng, B. *et al.* Effects of ultrasound on the morphology and properties of propylene-based plastomer/nanosilica composites. *Polym J* **43**, 91-96 (2011).

5 Lu, C. T., Weerasinghe, A., Maroudas, D. & Ramasubramaniam, A. A Comparison of the Elastic Properties of Graphene- and Fullerene-Reinforced Polymer Composites: The Role of Filler Morphology and Size. *Sci Rep-Uk* **6**, doi:Artn 31735 10.1038/Srep31735 (2016).
